# Bilateral Spontaneous Perirenal Hemorrhage in an Acquired Cystic Kidney Disease Hemodialysis Patient

**DOI:** 10.1155/2012/178426

**Published:** 2012-09-16

**Authors:** Daigoro Hirohama, Hiroshi Miyakawa

**Affiliations:** Division of Nephrology, Showa General Hospital, 2-450 Tenjincho, Kodaira, Tokyo 187-8510, Japan

## Abstract

Acquired cystic kidney disease (ACKD) is a well-known late stage complication of chronic kidney disease. Cysts tend to grow with time on dialysis and could lead to malignant transformation, and intra- or perirenal hemorrhage is a rare complication of ACKD. Here we describe one case of bilateral spontaneous perirenal hemorrhage of ACKD in a 44-year-old man, on hemodialysis for 15 years. One was due to cyst rupture, and the other was due to aneurism rupture, both were controlled with transcatheter arterial embolization. In renal arteriography at the second rupture, we demonstrated extravasation from an aneurysm being present at the periphery of right renal artery. Several spontaneous perirenal hemorrhage cases were reported but its clinical information is limited, moreover, bilateral cases were extremely rare. Furthermore, to our knowledge, this is the first report of spontaneous perirenal hemorrhage caused by intraparenchymal renal artery aneurysm rupture in ACKD patients. We report this case because of its rarity and significance with respect to the complication of dialysis patients, review reported bilateral cases, and discuss some clinical characteristics.

## 1. Introduction

Acquired cystic kidney disease (ACKD) is a complication of end-stage renal disease (ESRD), the prevalence of which is related to dialysis duration. Intra- and pericystic bleeding and rupture with retroperitoneal hemorrhage are rare complications of ACKD. We report an extremely rare case with bilateral spontaneous perirenal hemorrhage (SPH), which sequentially happened within seven months. Moreover, we review reported bilateral cases and discuss some clinical characteristics.

## 2. Case Report

A 44-year-old man with ESRD caused by hypertensive nephrosclerosis on hemodialysis for 15 years was admitted to our hospital for sudden onset of severe left flank pain and nausea. The pain had started suddenly about 2 hours before and worsened rapidly to its peak such that he had never experienced before.

His past medical history included hyperthyroidism, bilateral subdural hematoma, and coronary heart disease. He got a coronary artery bypass grafting (CABG) 11 months before his admission. Since then, he took aspirin and warfarin daily.

**Figure 1:**
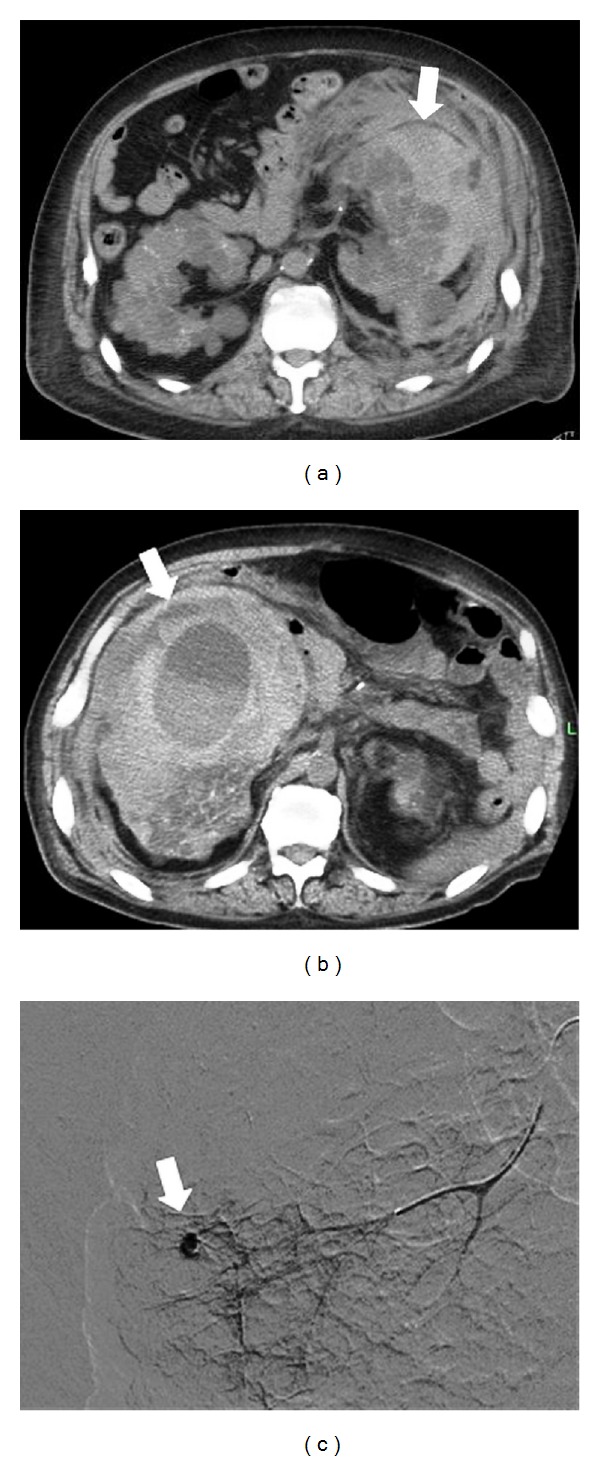
CT scan of the abdomen. (a) A large number of bilateral renal cysts were observed. A huge left perirenal hematoma (arrow) and (b) there was a large cyst including fluid with density of blood in right kidney, and a huge right perirenal hematoma (arrow). Renal arteriography during the right perirenal hemorrhage. (c) Extravasation from an aneurysm, being present at the periphery of right renal artery, was demonstrated (arrow).

 On admission, his blood pressure and pulse rate were 102/65 mmHg and 86 bpm, and he was symptomless except for mildly distended abdomen with severe tenderness over his left flank to back. Laboratory findings showed a low serum hemoglobin concentration 10.0 g/dL. In addition, PT-INR was slightly prolonged to 3.50. Computed tomography (CT) showed a huge hematoma extending from left kidney to perirenal and left retroperitoneal space. The scan also showed multiple cysts in both kidneys (Figure 1(a)). 

 Although, fluid resuscitation as well as prothrombin complex concentrates (PCC) for reversal of anticoagulation was initiated, his blood pressure declined to shock. In renal arteriography, we saw very mild extravasation at the periphery of left renal artery. We emergently performed transcatheter arterial embolization (TAE). Shortly after TAE, he recovered completely. 

 Seven months later, he was again admitted to the hospital because of fatigue and weakness. His blood pressure had been well controlled after discharge. On admission, no fever, vomiting, or other symptoms were present. His blood pressure and pulse rate were 92/60 mmHg, 102 bpm. His abdomen was mildly distended and mild tender to palpation in the right middle and lower quadrants. Laboratory findings showed a severe low serum hemoglobin concentration 5.3 g/dL, prolonged PT-INR 7.05, under anticoagulation therapy with warfarin. The CT scan showed a large cyst and a huge right perirenal hematoma (Figure 1(b)). The cyst, which was not found at the previous episode, showed fluid retention with density of blood. This time, in renal arteriography, we demonstrated aneurismal bleeding at the periphery of the right renal artery (Figure 1(c)). We again emergently perfomed TAE. After TAE, retroperitoneal hemorrhage remitted and he recovered completely in almost the same time course as the previous episode. 

 At outpatient review after 3 months of his discharge, the CT scan showed the contraction and absorption of retroperitoneal hemorrhage. We could not demonstrate any other cause of perirenal hemorrhage including renal cell carcinoma. He continues hemodialysis without any bleeding sign or symptom.

## 3. Discussion

 Spontaneous perirenal hemorrhage has been reported infrequency in the literature, where dialysis patients are often excluded and most frequent etiologies are renal cell carcinoma, angiomyolipoma, and vascular disease. Among dialysis patients, ACKD has been recognized to be the most frequent cause. In the past, there have been only 4 documented cases of spontaneous bilateral perirenal hemorrhage in ACKD dialysis patients [1–4] (Table 1). All the cases were men, etiologies of ESRD were hypertensive nephrosclerosis in 4 of 5 cases (80%), and the causes of bleeding were due mainly to the rupture of renal cyst.

 Interestingly, this is the first report of perirenal hemorrhage caused by intraparenchymal renal artery aneurysm rupture in ACKD patients. The large cyst including fluid with density of blood, which was not found at the previous episode, was likely to be formed by the intracystic extravasation from aneurysm. The incidence of renal arterial rupture in ESRD patients has not been fully defined. The mechanism of the formation of renal artery aneurysm is unknown. Mechanical factor from growing cysts, uneven distribution of oxygen, degenerative vascular change by amyloid deposition could contribute to some extent.

 Men and African-American population are more likely to develop ACKD. Also, kidney volumes are bigger and cyst size increases faster in this population [5]. There was a tendency for the rate of increase in ACKD to be larger in young males [5]. Spontaneous cyst rupture in the absence of trauma or infection is likely to occur because of increased intracystic pressure, which could reflect alterations in the dynamics of the cyst fluid [6], or massive intracystic bleeding supported by repeated systemic anticoagulation during dialysis procedures [7]. Furthermore, the risk for major bleeding episodes in hemodialysis patients increases significantly while on aspirin and/or warfarin [8], or on antiplatelet agents used in combination [9]. Recent review described that among SPH 95% are on hemodialysis rather than peritoneal dialysis and more than 90% received some kind of anticoagulation [10]. Since the rate of cardiovascular complication is high among patients on dialysis, those who take both antiplatelet and anticoagulant agents such as present case have been increasing. Hereafter, similar major bleeding complication such as SPH in dialysis patients might be expected to increase. 

 In conclusion, it is important to consider the possibility of spontaneous perirenal hemorrhage whenever clinician face acute abdominal symptom with low hemoglobin in dialysis patients. Antiplatelet and anticoagulant agents might increase the risk of renal rupture. Further study might contribute to the deeper understanding of the risk of this potentially fatal complication of ACKD.

## Figures and Tables

**Table 1:** Spontaneous bilateral perirenal hemorrhage in ACKD dialysis patients.

	Case 1	Case 2	Case 3	Case 4	Our case
First Author [Ref]	^ #^Minar et al. [1]	Carlson et al. [2]	Borràs et al. [3]	Ku et al. [4]	
Age, sex	35/M	48/M	37/M	46/M	44/M
Race	N.D.	N.D.	Black	N.D.	Yellow
HD/PD	HD	HD	PD (*5 years: HD)	HD	HD
Duration of dialysis	5 years	N.D.	5.5 years	8 years	15 years
Primary disease of ESRD	CGN	Hypertensive nephrosclerosis	Hypertensive nephrosclerosis	Hypertensive nephrosclerosis	Hypertensive nephrosclerosis
Renal Biopsy	N.D.	N.D.	Yes	N.D.	No
Past history	N.D.	CLL	CH-B	N.D.	CABG, Hypothyroidism
Time interval between bilateral perirenal hemorrhage	2 months	4 months	21 months	1 month	6 months
Etiology					
1st rupture	N.D.	Cyst rupture	Cyst rupture	**N.D.	Cyst rupture
2nd rupture	N.D.	Cyst rupture	Cyst rupture	**N.D.	Intraparenchymal renal artery aneurysmal rupture
Hemoperitoneum	N.D.	No	Yes	No	No
Antiplatelet agent	N.D.	N.D.	N.D.	N.D.	Aspirin, Cilostazol
Anticoagulant agent	N.D.	N.D.	N.D.	N.D.	Warfarin
Therapy					
1st rupture	N.D.	***Emergency surgery	Nephrectomy	Nephrectomy	TAE
2nd rupture	N.D.	TAE + Nephrectomy	Nephrectomy	TAE	TAE
Outcome	N.D.	Alive	Alive	Alive	Alive

N.D.: no data, HD: hemodialysis, PD: peritoneal dialysis, ESRD: end-stage renal disease, CGN: chronic glomerulonephritis, ACDK: acquired cystic kidney disease, CLL: chronic lymphocytic leukemia, CH-B: chronic hepatitis B, CABG: coronary artery bypass grafting, TAE: transcatheter arterial emboli.

^#^Article in Germany, abstract only.

*He was first treated with HD for 5 years.

**Intrakidney contrast extravasation suggestive of active bleeding was found.

***Disrupted fragments of the kidney were removed, and the renal pedicle was controlled with clamps suture ligated.
